# Botulinum Toxin Modulates Posterior Parietal Cortex Activation in Post-stroke Spasticity of the Upper Limb

**DOI:** 10.3389/fneur.2019.00495

**Published:** 2019-05-09

**Authors:** Tomáš Veverka, Pavel Hok, Pavel Otruba, Jana Zapletalová, Barbora Kukolová, Zbyněk Tüdös, Alois Krobot, Petr Kaňovský, Petr Hluštík

**Affiliations:** ^1^Department of Neurology, Palacký University and University Hospital, Olomouc, Czechia; ^2^Department of Biophysics, Biometry and Statistics, Palacký University and University Hospital, Olomouc, Czechia; ^3^Rehabilitation Centre, Hrabyně, Czechia; ^4^Department of Radiology, Palacký University and University Hospital, Olomouc, Czechia; ^5^Department of Physiotherapy, Palacký University and University Hospital, Olomouc, Czechia

**Keywords:** stroke, spasticity, botulinum toxin, functional magnetic resonance imaging, neuronal plasticity, motor imagery

## Abstract

Post-stroke spasticity (PSS) is effectively treated with intramuscular botulinum toxin type A (BoNT-A), although the clinical improvement is likely mediated by changes at the central nervous system level. Using functional magnetic resonance imaging (fMRI) of the brain, this study aims to confirm and locate BoNT-A-related changes during motor imagery with the impaired hand in severe PSS. Temporary alterations in primary and secondary sensorimotor representation of the impaired upper limb were expected. Thirty chronic stroke patients with upper limb PSS undergoing comprehensive treatment including physiotherapy and indicated for BoNT treatment were investigated. A change in PSS of the upper limb was assessed with the modified Ashworth scale (MAS). fMRI and clinical assessments were performed before (W0) and 4 weeks (W4) and 11 weeks (W11) after BoNT-A application. fMRI data were acquired using 1.5-Tesla scanners during imagery of finger-thumb opposition sequences with the impaired hand. At the group level, we separately modeled (1) average activation at each time point with the MAS score and age at W0 as covariates; and (2) within-subject effect of BoNT-A and the effect of time since W0 as independent variables. Comprehensive treatment of PSS with BoNT-A significantly decreased PSS of the upper limb with a maximal effect at W4. Task-related fMRI prior to treatment (W0) showed extensive activation of bilateral frontoparietal sensorimotor cortical areas, bilateral cerebellum, and contralesional basal ganglia and thalamus. After BoNT-A application (W4), the activation extent decreased globally, mostly in the bilateral parietal cortices and cerebellum, but returned close to baseline at W11. The intra-subject contrast revealed a significant BoNT-A effect, manifesting as a transient decrease in the activation of the ipsilesional intraparietal sulcus and superior parietal lobule. We demonstrate that BoNT-A treatment of PSS of the upper limb is associated with transient changes in the ipsilesional posterior parietal cortex, possibly resulting from temporarily altered sensorimotor upper limb representations.

## Introduction

Post-stroke spasticity (PSS) is a major sequelae among stroke survivors (1) with an estimated prevalence of 19–42.6% (2, 3). Clinically relevant PSS may interfere with voluntary movement and frequently causes deterioration in manual dexterity, mobility, walking, and hygiene (2). PSS of the upper limbs is currently treated with botulinum toxin type A (BoNT-A), which is an effective and safe therapeutic agent to improve function of the affected limb (4–6). BoNT-A treatment has been shown to relieve pain, enhance the effects of physiotherapy, improve performance in activities of daily living, and decrease the burden of caregivers (2). Over the last decade, there has been growing evidence that besides the well-known neuromuscular junction site of action, BoNT-A acts centrally. Whereas, direct effect on distant central circuits via retrograde transport and transcytosis in humans is still under debate (7), the central effects have been mostly ascribed to indirect changes due to plastic rearrangement subsequent to modulation of sensory input (8). BoNT-A likely relieves focal PSS by promoting dynamic changes at multiple levels of the sensorimotor system, presumably including the cerebral cortex. It has been suggested that BoNT-A acts on intrafusal as well as well as extrafusal fibers, thereby altering abnormal sensory input to the central nervous system via Ia afferent fibers (8, 9), which is likely the mechanism by which intramuscular BoNT-A injection induces cortical reorganization. The theory of central (remote) BoNT-A effects was first reported in electrophysiological studies of focal dystonia (10, 11). In dystonic disorders, one application of BoNT has been reported to be associated with even more pronounced microstructural gray matter changes in the frontal cortex, namely, primary motor cortex and pre-supplementary motor area (12). There have been several reports of the neuroanatomical correlates of BoNT-A-related relief of PSS using functional magnetic resonance imaging (fMRI) (13–16). However, the studies were conducted with small sample sizes; they differ in their activation tasks, and other methodological aspects. This makes direct comparison between the studies difficult. Patients with prominent upper limb spasticity indicated for BoNT-A treatment often have severe hand weakness, precluding the use of real hand movement. Motor imagery is feasible for severely affected patients and the sensorimotor representations may be preserved even in chronic paralysis (17). Motor imagery has been used widely in post-stroke paralysis, both as a functional neuroimaging probe sensitive to motor network abnormalities during stroke recovery (18) and as a motor training strategy (19). To our knowledge, our pilot study is the only one employing motor imagery to investigate cortical activation changes associated with PSS relief due to BoNT-A treatment (20). Using a longitudinal design, we expected that BoNT-A-induced change in afferent drive (8, 9) will be reflected in modulation of somatosensory cortical processing in the parietal areas (20). Even though our results showed several areas of change in the sensorimotor network over time, no regions showed transient effects following the course of dynamic changes in clinical spasticity. Therefore, the aim of the present longitudinal study was to identify BoNT-A-related patterns of cerebral cortex activation during motor imagery in a more representative cohort of patients with moderate to severe PSS of the upper limbs.

## Methods

The study protocol is described in our previous report (20). The following text summarizes the methodology and highlights differences particular for the present study.

### Patients

The study protocol was approved by the local Institutional Ethics Committee and conducted in accordance with the tenets of the Declaration of Helsinki. All subjects submitted written consent before participation in this study. The study cohort was limited to 30 right-handed chronic stroke patients (15 males and 15 females; median age, 65 years) with clinically relevant PSS of the upper limbs. Ischemic lesions were subcortical and corticosubcortical within the territory of the middle cerebral artery. The median time from stroke onset to study entry was 9 (range, 3–139) months. Exclusion criteria were: time after stroke of < 3 months; PSS not exceeding a score of 1 on the modified Ashworth scale (MAS) (21); history of BoNT-A application or drug affecting muscle hypertonus intake; severe cognitive deficit or depression, as assessed with the Mini Mental State Exam (22) and Zung Self-Rating Depression Scale (23), respectively, which could affect cooperation during the study protocol; and general MRI exclusions and contraindications. The patients' characteristics are listed in Table 1.

**Table 1:** Demographic and clinical characteristics.

**Patient**	**Stroke onset to W0 (months)**	**Lesion**	**Affected hand**	**mRS**	**BI**	**NIHSS**	**MMSE**	**Zung (SDS index)**	**mMRC (WF/WE)**	**mMRC (FF/FE)**	**Global MAS W0**	**Global MAS W4**	**Global MAS W11**
1	7	Thalamus, IC, BG	L	3	90	5	30	34	1+/0	1+/1+	3	1.5	2.5
2	3	BG, IC	R	3	85	8	29	30	0/0	0/0	3	1	2.5
3	3	BG, insula, thalamus, FT	L	4	40	9	19	45	0/0	0/0	3	2	3
4	6	Thalamus, IC, insula	L	4	45	7	23	65	1/0	1/0	3	1.75	2.5
5	5	Thalamus, IC	R	4	60	10	22	49	0/0	0/0	3	2	3
6	83	Insula, FP	R	2	95	4	29	39	2/1	2/1	2	1	2
7	6	Insula, BG, FT	R	3	70	8	N/A	63	0/0	0/0	2	1.25	1.75
8	11	thalamus, IC, BG	L	3	80	5	29	64	1/0	0/0	2.5	1.5	2.5
9	9	thalamus, BG, FT, insula	L	3	75	7	29	34	0/0	0/0	3	1.75	3
10	4	BG, insula, thalamus	R	3	80	6	27	48	0/0	0/0	2	1	2
11	32	BG, IC	L	3	70	5	28	59	2/1	3/1	2	1	2
12	64	Insula, FT	R	2	95	6	18	50	2/2	2/1+	2	1.25	2
13	23	BG, insula, FT	R	3	65	9	N/A	43	0/0	0/0	3	2	2
14	7	IC, F	R	2	100	3	25	40	3/3	3/2+	1.5	1.25	1.25
15	10	Thalamus,IC, insula	R	3	85	5	29	49	1/1	2/1	2	1	2
16	4	Thalamus, BG, insula	R	4	50	9	N/A	54	0/0	0/0	1.75	1.75	2
17	19	Thalamus,IC	L	3	75	7	30	53	0/0	0/0	3	1.25	2
18	28	Insula, BG, FT	L	4	60	11	20	34	0/0	0/0	2.5	1.25	1.75
19	9	Thalamus, BG, FT, insula	R	3	90	10	N/A	44	0/0	0/0	1.75	1	2
20	7	BG,IC	L	3	90	6	30	70	1/0	1/0	2	0.5	2
21	9	BG, insula, FT	L	4	65	5	28	65	0/0	0/0	2	1.5	2
22	139	FTP, BG	L	3	90	4	27	44	0/0	0/0	2.5	1.5	2.25
23	9	IC, BG	R	3	85	8	N/A	60	0/0	0/0	2.5	1.5	2
24	9	F, insula	R	3	90	9	30	50	0/0	0/0	2.5	2	2.5
25	4	FTP	L	3	80	5	30	44	3/2	3/2	3	1.75	2.5
26	76	BG, IC, F	R	3	95	4	26	59	3/2	3/2	2.5	1.75	2.5
27	10	BG, FT	R	3	85	6	30	54	2/0	2/0	2.5	2	1.75
28	14	BG, IC, F	R	3	85	7	30	51	2/1	2/1	2	2	2
29	43	F, insula	L	2	100	1	29	73	4/3	3/3	2	0.5	0.5
30	38	BG, IC, FT	L	4	65	9	29	53	1/0	1/0	3	3	3

*L, left; R, right; BG, basal ganglia; IC, internal capsule; F, frontal lobe; T, temporal lobe; P, parietal lobe; NIHSS, NIH stroke scale; mRS, modified Rankin Scale; mMRC, modified MRC scale; BI, Barthel index; WE, wrist extensors; WF, wrist flexors; FE, finger extensors; FF, finger flexors; MAS, Modified Ashworth scale; N/A, not applicable (MMSE score could not be interpreted because of the presence of expressive aphasia)*.

### Clinical Evaluation

All subjects were clinically evaluated just before BoNT-A injection (week 0, W0), then 4 weeks (W4) and 11 weeks (W11) later. Longitudinal within-subject design of the study partially overcomes the lack of a control group. Here, each patient serves as his/her internal control. PSS was evaluated with the MAS at each visit. The MAS was used to score the fingers and wrists separately, and the values were averaged (global MAS score). The MAS rater was blinded to the therapy and the recruitment to the present study. For statistical analysis, a MAS score of 1+ was recorded as 1.5. Further clinical investigations performed at study entry included the modified Medical Research Council scale (24) to test upper extremity strength, the National Institutes of Health stroke scale (25) to assess neurological impairment, and the Barthel Index (26) and the modified Rankin Scale (27) to assess disability.

### Treatment

Enrolled patients received BoNT-A injections into the muscles of the affected upper limb at W0, which was followed by a dedicated physiotherapy protocol. The injections were performed under electromyographic guidance (Medtronic Keypoint; Alpine Biomed ApS, Skovlunde, Denmark), preferably with electrical stimulation to localize the muscles to be treated. The following muscles were always injected: flexor carpi ulnaris, flexor carpi radialis, flexor digitorum superficialis, and flexor digitorum profundus. Each muscle was consistently injected with a fixed dose of 50 U of BoNT-A (BOTOX^®^; Allergan, Inc., Irvine, CA, USA) in accordance with current recommendations (4). Rehabilitation was started several days after the BoNT-A injection (W0). Initial inpatient physiotherapy for 2–4 weeks was followed by outpatient therapy until the third clinical and fMRI evaluation (total of 11 weeks). Patients underwent daily physiotherapy sessions for 1 h on workdays, i.e., five times per week. Individual kinesiotherapy included posture-locomotion training toward restitution of bipedal posture and gait, motor recovery of the girdles and trunk using elements of the Bobath concept, proprioceptive neuromuscular facilitation, respiratory physiotherapy, reflex and myofascial techniques, anti-spastic positioning, occupational therapy, and training of independence in activities of daily living. Proper adherence to the physiotherapy protocol was checked at each examination throughout the study period (28).

### fMRI Data Acquisition

fMRI examinations were performed during the clinical evaluations at W0, W4, and W11 using a 1.5-Tesla scanner (Avanto or Symphony; Siemens Healthineers, Erlangen, Germany) equipped with a standard head coil. Whole-brain blood oxygenation level-dependent (BOLD) fMRI data (${\text{T}}_{2}^{*}$-weighted echo-planar imaging; 30 slices, 5 mm thick; repetition time, 2,500 ms; 144 volumes; repeated twice) were acquired during imagery of finger movements with the impaired hand. A high resolution T_1_-weighted structural image was acquired using Magnetization-Prepared Rapid Gradient-Echo (MP-RAGE) sequence for anatomical reference. Before the first fMRI examination, each subject practiced the sequential finger-thumb opposition task with the non-paretic hand at the rate of approximately 1 movement per second over several repetitions and then was asked to imagine performing the same movement with the impaired fingers together with kinesthetic feeling. Before the follow-up fMRI examinations, we checked the correct performance of the task with the unimpaired hand. The only purpose of pre-imaging practice was to allow stable performance across the study. Inside the bore of the scanner, the task was performed with eyes closed, instructions to start and stop task performance were signaled verbally (start/stop) in MR-compatible headphones. In a block paradigm, imagery of finger movement alternated with rest (15 s). Each experimental run consisted of 12 repetitions of the same imagery-rest block pairs, for a total of 6 min. Each participant had two experimental runs with the impaired hand. The experimental paradigm has been already used and published in Veverka et al. (20).

### Analysis

fMRI data of patients with right-sided lesions were swapped to match the left-sided lesions (29, 30). Next, a previously published preprocessing pipeline was applied (16). Functional images were registered to high resolution structural images and normalized to the standard space template using linear and non-linear algorithms, respectively (31). At the final pre-processing stage, residual motion-related signals were automatically removed using independent component analysis-based automatic removal of motion artifacts (32).

Statistical analysis of the functional time-series was conducted using general linear modeling with local autocorrelation correction (33). The boxcar function of the block design was convolved with a canonical hemodynamic response function (34) and a temporal derivative to account for the relative slice-dependent time shift rather than slice-wise time-course interpolation (35). Furthermore, several nuisance regressors were obtained from the functional data of each subject and added to the general linear model: six motion parameters, one signal from the white matter, and one from the cerebrospinal fluid.

After first-level processing, repeated measures from the same session were averaged for each subject using a middle-level analysis. Group statistical analyses were performed using stage 1 of the improved linear model for fMRI of the brain (36, 37). At the group level, (1) the average activation was separately modeled at each time-point with the MAS and age at W0 as linear covariates; and (2) the within-subject effect of BoNT-A [(W0 + W11)/2 – W4)] and linear effect of time from W0 were assigned as independent variables. The model was designed to separate the transient effect of BoNT from the presumed linear effect of physiotherapy. The resulting statistical maps were thresholded using clusters at *p* < 0.05 (family-wise error-corrected) formed at (1) *Z* > 3.0 for average activation and (2) *Z* > 2.3 for within-subject effects (family-wise error-corrected using Gaussian random field theory) (38). Within-subject effects were additionally Bonferroni-corrected for the number of contrasts.

Clinical data were analyzed using IBM SPSS Statistics for Windows, version 22.0 (IBM Corp., Armonk, NY, USA). The Wilcoxon signed-rank test with Bonferroni correction was used to compare global MAS scores from W0, W4, and W11. A probability (*p*) value of < 0.05 was considered statistically significant.

**Figure 1:**
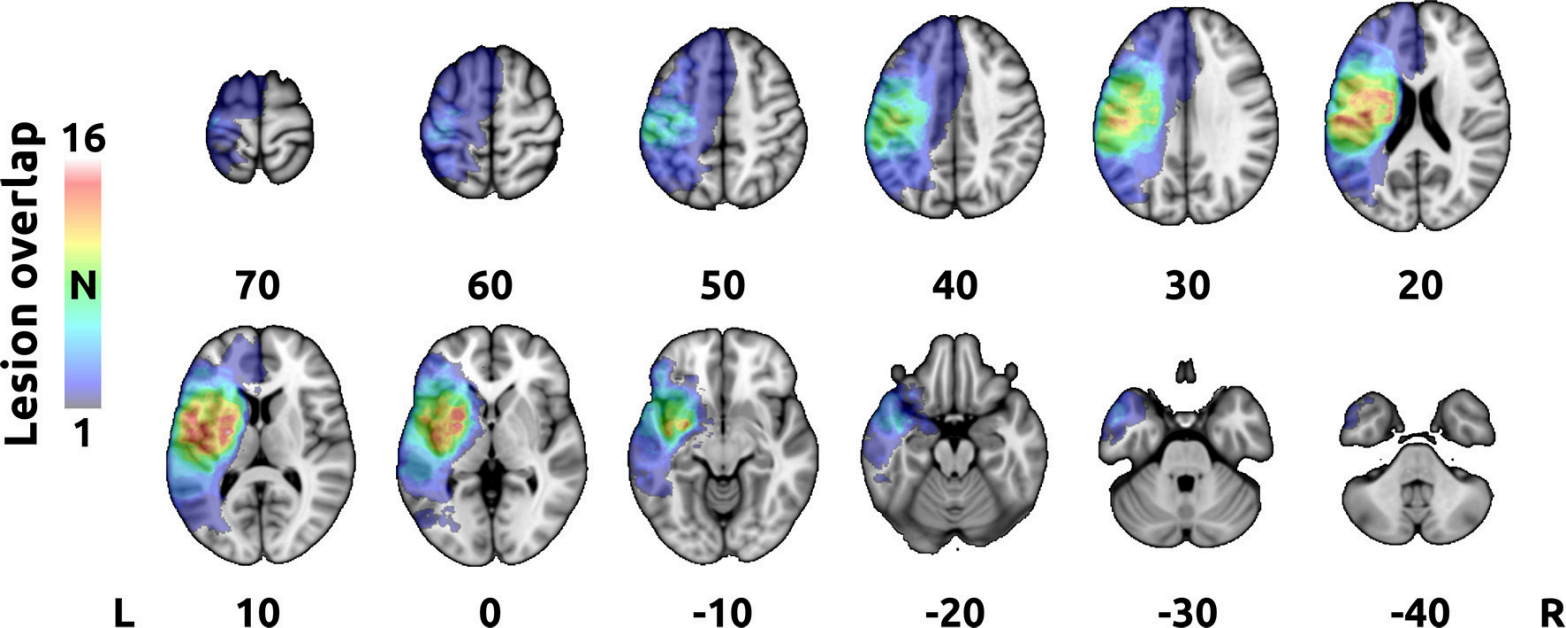
Group-wise stroke lesion map. The color-spectrum overlay represents an unthresholded sum of normalized individual stroke lesion masks on top of an MNI 152 standard space T_1_-weighted template. Cold colors (blue) indicate low overlap (~1–2 subjects), hot colors (yellow-red-white) indicate frequent overlap (up to 16 subjects). Right is right according to neurological convention.

Additionally, a map of stroke lesions was created to visualize the overall volume of the affected tissue. First, T_1_-hypointense stroke lesions were delineated semi-automatically on the high-resolution structural images using interactive intensity-based volume segmentation in each individual. The resulting binary masks were manually corrected for errors by PaH. Next, masks with right-sided lesions were swapped to match the left-sided lesions and all masks were transformed into 1-mm MNI 152 standard space using a non-linear transformation (31). Finally, sum of all masks was created to provide a group-wise lesion map (Figure 1).

## Results

### Clinical

**Figure 2:**
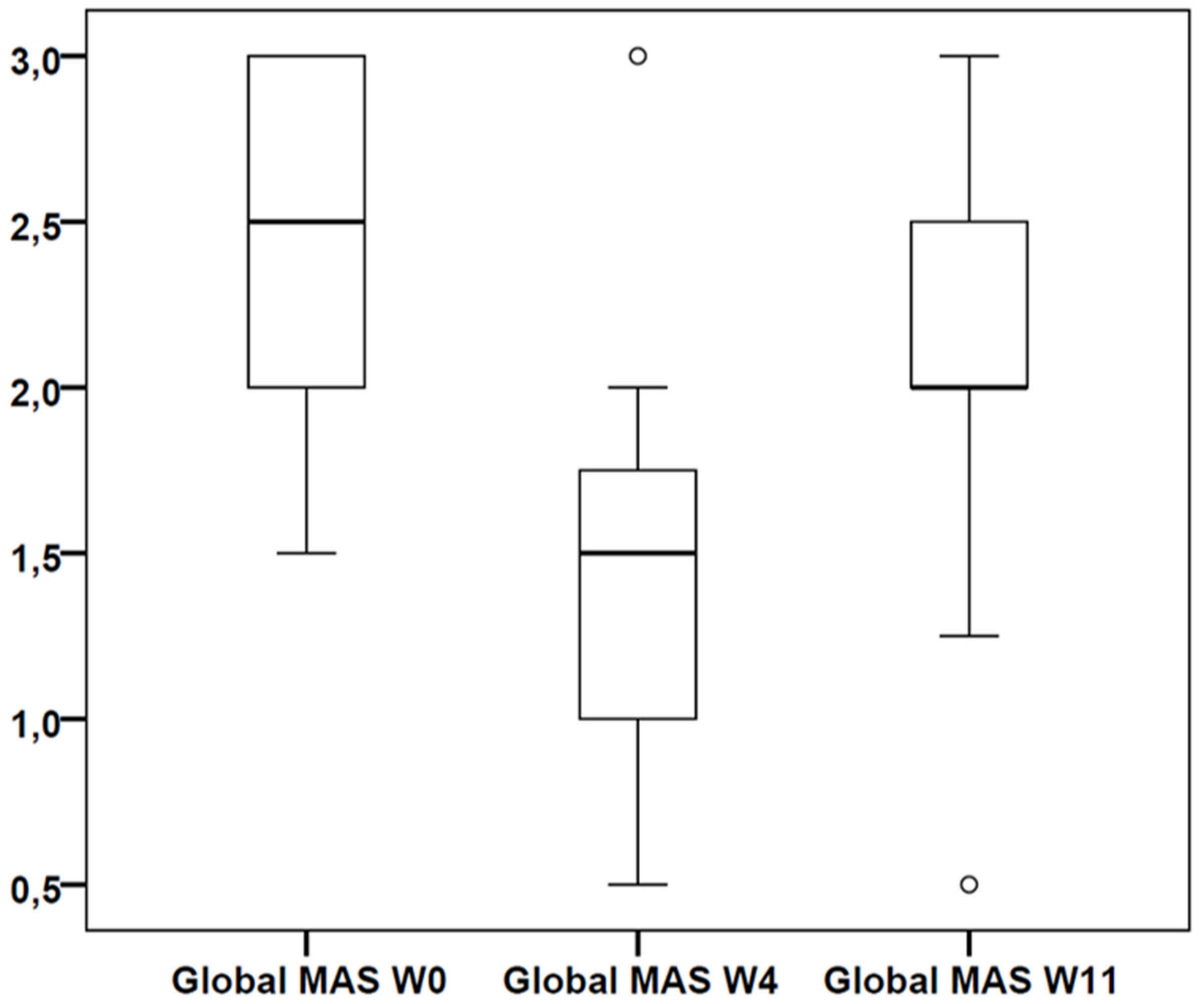
Effect of BoNT-A treatment on global MAS scores. The edges of the box represent the 25th and 75th percentiles, the horizontal thick line inside the box represents the median, and the whiskers represent the maximum and minimum values.

Comprehensive treatment with BoNT-A and subsequent physiotherapy significantly decreased PSS of the upper limb with the maximal effect at W4 (*p* < 0.0001, Wilcoxon signed-rank test). There were significant differences in global MAS between W0 and W11 (*p* = 0.006) and between W4 and W11 (*p* < 0.0001, Wilcoxon signed-rank test). The median global MAS scores were 2.50 at W0 (interquartile range (IQR) = 2.0–3.0), 1.50 at W4 (IQR = 1.0–1.75), and 2.00 at W11 (IQR = 2.0–2.5). The data are presented in a box plot in Figure 2. The MAS scores for each subject are listed in Table 1. The overlap of stroke lesions in all participants is provided in Figure 1.

### Functional Imaging

**Figure 3:**
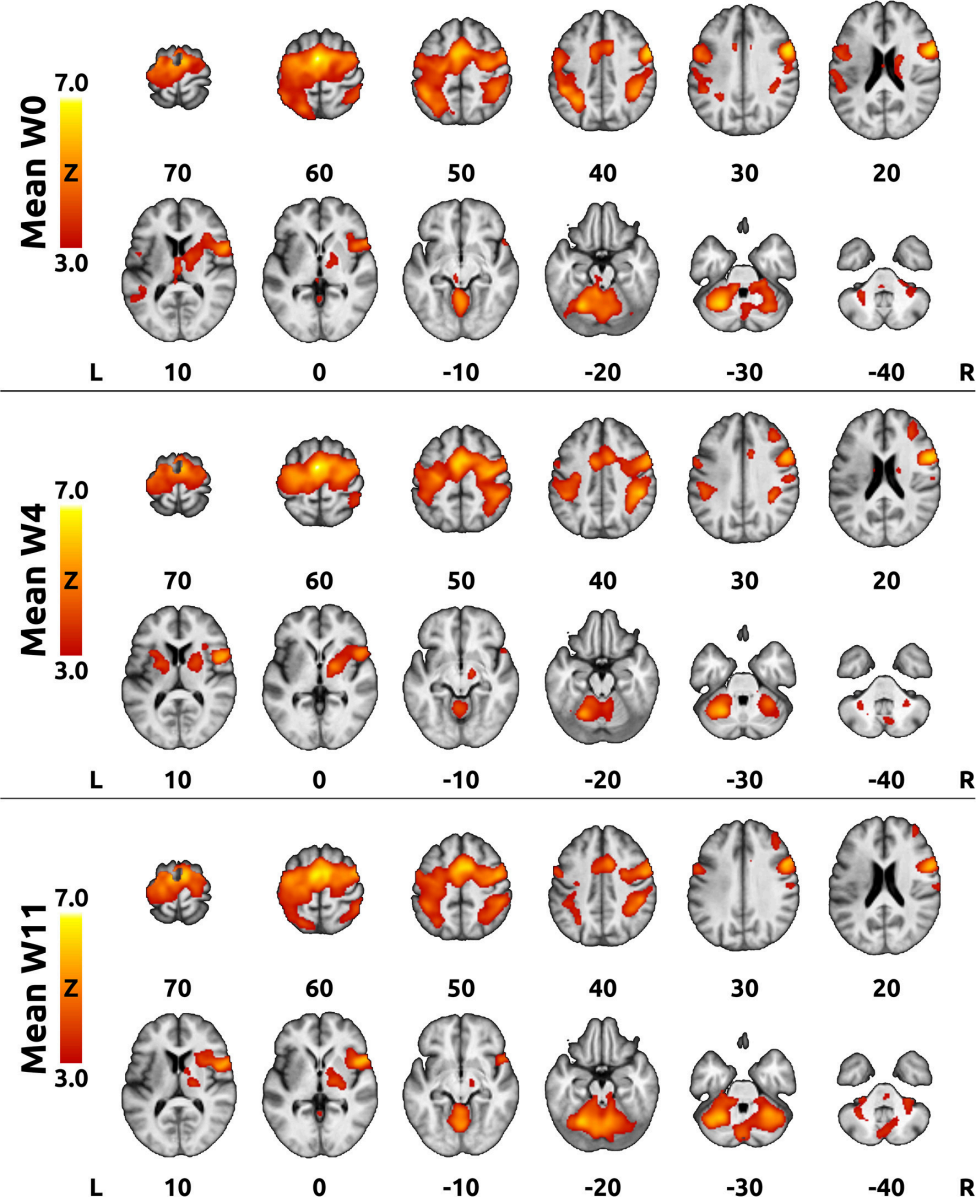
Mean activation at each time point. The red-yellow color overlays represent Z statistical maps thresholded cluster-wise at *Z* > 3.0 and cluster-wise corrected *p* < 0.05. An averaged T_1_-weighted image was used as a background. The top panel shows the mean activation at week 0 (W0), the middle panel shows activation at 4 weeks after BoNT-A injection (W4), whereas the bottom panel displays activation at 11 weeks after BoNT-A injection (W11). Right is right according to neurological convention.

Task-related fMRI prior to treatment (W0) showed extensive activation of the bilateral frontoparietal sensorimotor cortical areas, bilateral cerebellum, and contralesional basal ganglia and thalamus, with peak activation in the supplementary motor area (SMA), bilateral intraparietal sulci (IPS), contralesional ventrolateral premotor cortex, and ipsilesional anterior and posterior cerebellar hemispheres. After BoNT-A application (W4), the activation extent decreased globally, mostly in the bilateral parietal cortices and cerebellum, but returned close to baseline at W11 (see average activation maps in Figure 3).

**Figure 4:**
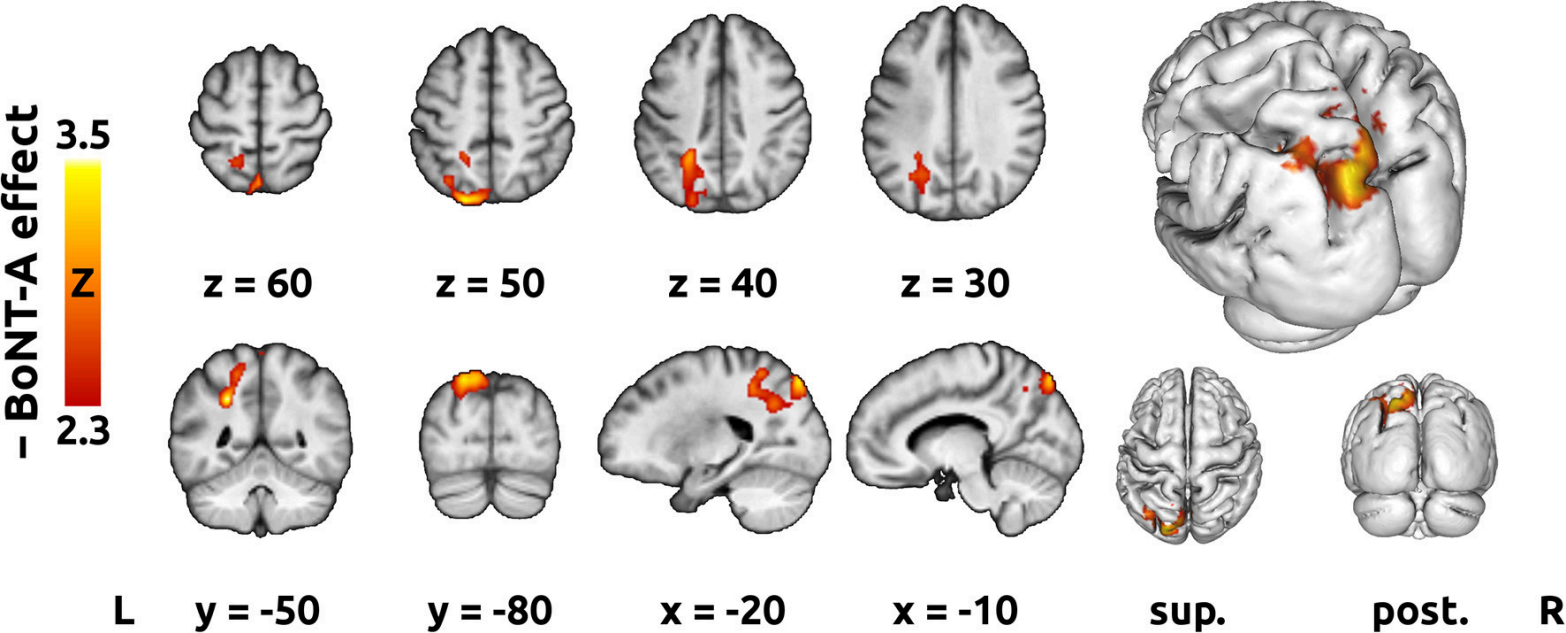
Treatment-related activation change. The red-yellow color overlay represents a Z statistical map of the negative BoNT effect (i.e., transient activation decrease at W4), thresholded cluster-wise at *Z* > 2.3 and cluster-wise corrected *p* < 0.05. On the right, the statistical map has been superimposed onto a reconstructed average brain surface. sup., superior view; post., posterior view. Remaining conventions (see Figure 2).

The intra-subject contrast revealed a significant BoNT-A effect, which manifested as transient decreases in activation of the ipsilesional superior parietal lobule (SPL) and IPS (Figure 4 and Table 2). No consistent activation changes related to time since W0 were observed.

**Table 2:** Treatment-related activation differences—list of local maxima.

**Contrast**	**Anatomical atlas labels^a^**	**Cytoarchitectonic atlas labels^a^**	**Volume [cm^**3**^]**	**Cluster *p (corrected for multiple comparisons)***	**Z_**max**_**	**MNI coordinates of local maxima^b^** **[x,y,z (mm)]**
Negative BoNT-A effect: Cluster 1	51.5% L Lateral Occipital Cortex, superior division 21.7% L Precuneous Cortex 20.2% L Superior Parietal Lobule	29.2% L Superior Parietal Lobule 7A 20.2% L Superior Parietal Lobule 7P 14.1% L Anterior intra-parietal sulcus hIP3 13.8% L Anterior intra-parietal sulcus hIP1	13.76	0.032	3.49	−24, −50, 36 −18, −82, 50 −12, −82, 52 −4, −74, 56 0, −70, 58

*BoNT-A, botulinum toxin type A; L, left; MNI, Montréal Neurological Institute; Z_max_, maximum Z score*.

a *Anatomical and cytoarchitectonic labels are provided including the proportion of labeled voxels. Only labels consisting at least 5% of activated voxels are provided. Note that cytoarchitectonic labels do not cover the whole brain*.

b *Top five local maxima with the highest Z score are provided*.

## Discussion

The brain is continually reorganizing (39). Stroke triggers processes in the central nervous system aimed to promote post-stroke recovery (adaptive plasticity). Some of these processes may not be beneficial or can even worsen primary neurological impairment (maladaptive plasticity), such as the substantially negative impact of PSS on manual dexterity, mobility, and ultimately harmful effects on the patient's health-related quality of life (1, 2). From this point of view, effective treatment of PSS might not only diminish muscle hyperactivity, but could also replace maladaptive with adaptive plasticity.

The present study is an extension of our previous study of a smaller sample size (20) and provides new evidence supporting the theory of cortical reorganization after BoNT-A treatment. Imagery of sequential finger movement was used as an activation task in an fMRI experiment. Kinesthetic imagery activates highly similar cortical areas as actual movements (40, 41). A meta-analysis conducted by Hétu et al. (42) showed that motor imagery consistently recruits the large frontoparietal network besides the subcortical and cerebellar regions, and identified the following areas as involved in motor imagery: the inferior parietal lobule, SPL, dorsal premotor cortex, SMA, cerebellum, and Broca's area. It has been demonstrated that motor imagery is a feasible task for severely affected patients unable to perform an active motor task, although motor imagery is difficult to monitor (17, 20). However, pre-scan practice in our patients before the first fMRI was intensive and sufficiently long to ensure reliable performance, and correct memory of the task was checked before the follow-up fMRIs, to overcome this limitation. Additionally, each fMRI acquisition was checked separately for corresponding BOLD activations.

As expected, the combination of BoNT-A treatment and physiotherapy effectively alleviated PSS of the upper limb as reflected by the MAS score. There was a significant transient decrease in the global MAS score at W4, when the pharmacological peripheral effect of BoNT-A is assumed to be maximal, and a subsequent increase in the global MAS score at further follow-up (W11). In contrast to our previous BoNT-A-studies (15, 16, 20), there was a significant change in the global MAS score from baseline to W11, when BoNT-A is expected to wane from the neuromuscular junctions (43). Namely, some improvement of spasticity persisted by the end of the study, even though local BoNT-A effect should have disappeared. Although this novel finding might support the theory of persistent central reorganization after BoNT-A application, the effect of ongoing physiotherapy should also be considered.

Task-related fMRI prior to treatment showed extensive activation of the bilateral frontoparietal sensorimotor cortical areas, bilateral cerebellum, and contralesional basal ganglia and thalamus, with peak activation in the SMA, IPS, contralesional ventrolateral premotor cortex, and ipsilesional anterior and posterior cerebellar hemispheres. The prominent involvement of the premotor cortical areas and relatively minor activation of the primary motor cortices during motor imagery is consistent with previous observations both in healthy controls (41, 44–47) and stroke patients with motor deficits (18, 48).

Alleviation of PSS at W4 was associated with an apparent reduction in the extent of activation, mostly of the bilateral parietal cortices and cerebellum, but returned close to the original extent at W11. This finding is in agreement with our previous studies and other previously published fMRI studies uncovering cerebral correlations with PSS treatment (14–16, 20, 49–52). Extended task-related cortical activation probably represents a general response of the lesioned brain to increased proprioceptive afferent input associated with PSS (13, 14). The overall reduction in the extent of activation after treatment might reflect transient changes due to BoNT-A administration and/or physiotherapy.

A similar trend has been observed in the evolution of the extent of activation during stroke recovery (53–57). A vast motor task-related activation of the bilateral frontoparietal cortex early after stroke is followed by a decrease in the extent of activation and increase in laterality in recovering patients (29, 30). We assume that this phenomenon did not bias our results for several reasons. First, all enrolled subjects were severely affected and their capacity for motor improvement was strongly limited. Second, only chronic stroke patients were included, thus the time from stroke onset to study entry was sufficiently long (median 9 months) to assure the stability of clinical features and a hemodynamic response (29, 58).

To address the main aim of the study, an intra-subject contrast design was used to separate the specific BoNT-A effect from the longitudinal effects of time and/or ongoing physiotherapy. The transient effect associated with BoNT-A observed in our study manifested as a significant decrease in activation of the ipsilesional posterior parietal cortex (PPC), namely the SPL and the cortex surrounding IPS.

The PPC, that is, the entire parietal cortex behind the primary and secondary somatosensory cortices, is part of a broad anatomical network of frontoparietal association (multisensory) cortical areas, which encode the more abstract aspects of sensorimotor control processes (59–61). Several functional domains have been attributed to this network, for instance, the dorsal attention network that directs visual attention and short-term memory (60). The dorsal attention network partially overlaps with another functional network, namely the motor imagery network (42). Finally, the PPC is involved in the visual system, particularly in its dorsal stream (occipito-parietal cortex) (62, 63).

In general, the PPC is therefore involved in perception and processing of action-related information. More specifically, the PPC is recruited by sensory control of visuomotor actions, such as reaching, pointing, grasping, and eye movement (59, 63). In our previous work with a similar design, but a smaller sample size, IPS and SPL were among the areas showing significant reductions of the spatial extent of activation after BoNT-A treatment, but the contrast assigned to the specific BoNT-A effect did not reveal any areas of significant change in the magnitude of the local BOLD effect (20). The absence of BoNT-A-related effects in our pilot study might be attributed to the relatively small sample size, which reduced the overall statistical power to detect smaller treatment effects.

As suggested by the findings of the present study, decreased activation of the IPS and SPL after treatment reflects a change in internal representation of the subject's hand resulting from decreased inflow of proprioceptive information from the spastic limb. It has been previously demonstrated that brain activation during motor imagery is strongly influenced by the proprioceptive information related to the pre-existing configuration of the limbs (64). After successful treatment of PSS, the internal models (predictions) of the upper limb are likely to adapt to the newly reduced flow of afferent information, which could, in turn, reduce the occurrence of unnecessary fMRI activation during motor imagery, as was observed with actual movement (15). Similar effects on overt and imagined upper limb movement were observed in Parkinson disease patients before and after treatment with L-DOPA (65, 66). A theory of internal model utilization in motor imagery has been supported by a recent study conducted by Kilteni et al. (67), which concluded that motor imagery recruits the internal forward model to predict sensory consequences similarly to overt execution. Another study using magnetoencephalography suggested that kinesthetic feeling is subserved by an internal forward model located in the parietal cortex, particularly the cortex surrounding the IPS, highlighting its role in motor imagery (68). Moreover, a motor imagery study conducted by de Lange et al. (64) found that the PPC appears to incorporate afferent proprioceptive information into the motor plan.

Alternatively, we might speculate that in our group of severely affected poststroke patients, visual imagery may have prevailed over kinesthetic imagery at baseline, although the participants were asked to imagine the action with kinesthetic feeling. Studies of motor imagery in post-stroke patients suggest that for these subjects, it is very difficult to use either visual or kinesthetic imagery selectively (19, 69). Therefore, we assume that the severely affected subjects enrolled in the present study employed a combination of both imagery strategies. Moreover, we did not design the experiment to discriminate between different aspects of motor imagery, because the Movement Imagery Questionnaire and similar alternative tests were beyond the abilities of the patients (70, 71). Due to the prominent role of the SPL and occipital regions in visual input processing (41, 72), the BoNT-A-related reduction of PPC activation at W4 might be interpreted as a lower engagement of the cortical areas attributed to visual imagery. From this perspective, it is possible that alleviation of PSS renders the contribution of visual strategy in motor imagery less prominent.

There were some limitations to this study that should be mentioned. First, we did not include a control group without BoNT-A treatment, which would have been optimal to separate transient effect of BoNT-A from more longitudinal effect of concurrent physiotherapy. For several years, BoNT-A treatment has been a recommended component of the complex therapy regimen for PSS (4, 5). Therefore, non-treatment would have been unethical. Although functional cortical changes observed in the present study could have been induced by both the BoNT-A treatment and the physiotherapy, we argue that the within-subject longitudinal design, with three successive assessments over 3 months, captures both the transient changes due to BoNT-A and the more slowly evolving changes in sensorimotor control due to ongoing physiotherapy and symptomatic therapy. This approach has been considered sufficient to address the main goal of the study—to uncover specific effect of BoNT-A in the studied population of chronic stroke patients. Finally, the heterogeneity in stroke location and the degree of cortical involvement limit the possibility of generalizing the results to the whole population of patients with upper limb PSS.

## Conclusions

Whole brain fMRI activation patterns during motor imagery in the course of BoNT-A treatment of upper limb PSS and further follow-up documented mostly transient changes in the ipsilesional PPC. Our results indicated that BoNT-A therapy modulated posterior parietal cortical activation in PSS even in chronic patients with severe hand weakness.

## Ethics Statement

The study protocol was approved by the local Institutional Ethics Committee and conducted in accordance with the tenets of the Declaration of Helsinki. All subjects submitted written consent before participation in this study.

## Author Contributions

TV and PeH: conceived and designed the experiments. TV, PeH, ZT, BK, and PO: performed the experiments. PaH and JZ: analyzed the data. TV, PaH, and PeH: interpretation of results. TV and PaH: wrote the paper. PK and AK: supervision.

### Conflict of Interest Statement

The authors declare that the research was conducted in the absence of any commercial or financial relationships that could be construed as a potential conflict of interest.
